# An Interpretable Double-Scale Attention Model for Enzyme Protein Class Prediction Based on Transformer Encoders and Multi-Scale Convolutions

**DOI:** 10.3389/fgene.2022.885627

**Published:** 2022-04-01

**Authors:** Ken Lin, Xiongwen Quan, Chen Jin, Zhuangwei Shi, Jinglong Yang

**Affiliations:** ^1^ College of Artificial Intelligence, Nankai University, Tianjin, China; ^2^ College of Computer Science, Nankai University, Tianjin, China

**Keywords:** enzyme class prediction, self-attention, multi-scale convolutions, double-scale attention, feature agreement

## Abstract

**Background** Classification and annotation of enzyme proteins are fundamental for enzyme research on biological metabolism. Enzyme Commission (EC) numbers provide a standard for hierarchical enzyme class prediction, on which several computational methods have been proposed. However, most of these methods are dependent on prior distribution information and none explicitly quantifies amino-acid-level relations and possible contribution of sub-sequences.

**Methods** In this study, we propose a double-scale attention enzyme class prediction model named DAttProt with high reusability and interpretability. DAttProt encodes sequence by self-supervised Transformer encoders in pre-training and gathers local features by multi-scale convolutions in fine-tuning. Specially, a probabilistic double-scale attention weight matrix is designed to aggregate multi-scale features and positional prediction scores. Finally, a full connection linear classifier conducts a final inference through the aggregated features and prediction scores.

**Results** On DEEPre and ECPred datasets, DAttProt performs as competitive with the compared methods on level 0 and outperforms them on deeper task levels, reaching 0.788 accuracy on level 2 of DEEPre and 0.967 macro-*F*_1_ on level 1 of ECPred. Moreover, through case study, we demonstrate that the double-scale attention matrix learns to discover and focus on the positions and scales of bio-functional sub-sequences in the protein.

**Conclusion** Our DAttProt provides an effective and interpretable method for enzyme class prediction. It can predict enzyme protein classes accurately and furthermore discover enzymatic functional sub-sequences such as protein motifs from both positional and spatial scales.

## 1 Introduction

Enzyme proteins are a type of functional protein that is necessary for biological metabolism *in vivo*. They accelerate (termed catalyze) chemical reactions in the cell, supplying reaction products and energy during metabolizing Berg et al. (2002).

In general, each enzyme specializes in one single reaction, making a unique attribution for this protein. According to this selectivity of enzymes and the type of catalyzed reactions, the International Union of Biochemistry and Molecular Biology Cornish-Bowden (2014) assigns standard enzyme commission (EC) numbers for enzyme proteins as a curator-friendly and machine-readable paradigm Dalkiran et al. (2018). An enzyme’s EC number consists of 4 levels of integer digits concatenated by periods. The first level (level 1) refers to one of the 6 main enzymatic classes (e.g., 1 for oxidoreductases and 6 for ligases) and levels 2 and 3 represent subclass and sub-subclass. The last level (level 4) stands for the substrate of the enzyme.

Enzyme protein class prediction is a hierarchical classification task. The task classifies enzyme and non-enzyme protein sequences at level 0 and further predicts the EC number of each enzyme protein by layer. In essence, enzyme class prediction infers the catalyst function of proteins. Classical methods apply one or more plain classifiers (e.g., KNN variants, Pepstats, and SPMap) to protein sequences (one-hot encoding) and biological prior features (e.g., position-specific scoring matrix PSSM and Functional domain FunD composition) for enzyme class prediction (Shen and Chou, 2007; Dalkiran et al., 2018). However, these methods require manually crafted and length-dependent features and extra evolutionary information from prior features.

Recently, deep learning predictors have been successfully applied to encode protein (Deng et al., 2015; Wang et al., 2015; Long et al., 2018) and classify enzymes Li et al., 2018; Gao et al., 2019; Strodthoff et al., 2020). Li et al. (2018) proposed an end-to-end classification model, DEEPre, which introduces CNN layers to select and strengthen raw features constructed from the input sequence directly. Inspired by the success of self-supervised approaches in natural language processing, Strodthoff et al. (2020) proposed a pre-trained AWD-LSTM (Merity et al., 2017) based deep language model to predict enzyme class. Compared to classical predictors, this model can make relatively accurate predictions only by raw sequences. Although the deep learning methods above construct high-level protein features well, they do not explicitly compute position-wise amino acid relationships and protein motif significance. There are two techniques that can overcome the problems above.

The first technique is the self-attention mechanism. This technique can explicitly catch the correlation between amino acids in protein sequences and has been applied to powerful sequential models in the recent decade. Self-attention directly quantifies the attention weight between each positional pair of feature vectors by their dot product (Gehring et al., 2017). Recently, Vaswani et al. (2017) proposed the Transformer, an epoch-making architecture that extends self-attention to a multi-head attention module. Then BERT (Devlin et al., 2018) brings up a pre-training language model using stacked bidirectional Transformer encoders. Both Transformer and BERT prove that self-attention is capable of capturing long-term dependencies in sequence without temporal delay.

Several works have shown the potential of self-attention–based models, especially Transformers, for modeling biological sequences (e.g., DNA and protein) and predicting molecular functions. Ji et al. (2021) pre-trained a BERT on k-mer DNA fragments and fine-tuned the model on a small sample of DNA sequences. The fine-tuned model predicts multiple functional DNA sites with high precision. Clauwaert et al. (2021) trained a Transformer-based network to locally annotate genomic sequences. They discovered that the attention heads of the Transformer successfully encoded the binding sites of transcription factors. Vig et al. (2020) experimented on pre-trained BERTs with multiple protein sequence datasets. They showed that BERTs can discover the folding structure and target binding sites of proteins. Rives et al. (2021) deployed a large number of raw protein sequences to train a self-supervised Transformer. They proved that the representation space of their model covers latent knowledge scaled from amino acid to protein homologs. The works above indicate from different perspectives that self-attention–based models are qualified for protein embedding and downstream enzyme class prediction tasks.

The second technique is the multi-scale convolution. Abundant functional and structural information of biological sequences are implicitly encoded in some variable-length patterns, termed motifs Debret et al. (2009). 1-D convolution is widely used to extract fixed-length pattern features of biological sequential data due to its fixed kernel size and shift-invariant property. Therefore, multi-scale convolutions (i.e., a group of convolutions with different kernel sizes) can capture patterns of multiple lengths including protein motifs.

In practice, some methods have been proposed to extract hierarchical local protein features with the multi-scale convolutions. Li and Yu (2016) extracted local representations from pre-processed protein features on different scales using convolutions of three kernel sizes. The local representations were concatenated and fed into the recurrent neural network to predict protein secondary structure. Elbasir et al. (2019) applied a multi-layer multi-scale CNN to simulate k-mer methods in biological sequence analysis. The CNN-encoded features emphasize amino acids whose contribution to protein crystallization is experimentally verified. Zeng et al. (2020) introduced textCNN Chen (2015) to the feature extraction process of protein–protein interaction site prediction. The textCNN designs a multi-scale convolution layer to capture features of multi-length sub-sequences. Jin et al. (2021) aggregated k-mer amino acids by multi-scale convolution and merged all scale-wise features by self-attention. Their experiments demonstrated that multi-scale convolution enriches the embedded features for protein crystallization prediction. The applications above show that multi-scale convolutions have some similarity to biological methods in extracting local sub-sequential information.

In this study, we construct a sequence classification model based on a double-scale attention mechanism, called DAttProt, for enzyme class prediction. DAttProt is the first model applying Transformer encoders and BERT-styled Masked LM Devlin et al. (2018) pre-training progress to the enzyme class prediction task. Our main contributions are as follows:

1. Self-supervised pre-training for sequence representation: We pre-train Transformer encoders by protein sequences from the Swiss-Prot Bairoch and Apweiler (2000) database. The pre-training procedure is self-supervised and aimed at finding inner correlations and representations of amino acids.

2. A feature agreement algorithm for multi-scale features: We deploy multi-scale convolutions to extract features from multiple semantic levels of adjacent amino acids. A feature agreement algorithm based on vector dot-product is designed to allocate attention weights to multi-scale feature aggregation and positional enzyme class scores summary.

3. Interpretable double-scale matrix: Additionally, the feature agreement algorithm provides a probabilistic and interpretable weight matrix on both positional and special scales. This double-scale attention matrix focuses on the position and length of key motifs of protein enzyme catalysis.

We compare our DAttProt (with two depths: 3-layer and 6-layer) with “DEEPre” (to distinguish the DEEPre method from the DEEPre dataset, we use “DEEPre” to represent the DEEPre method, similarly hereinafter), “ECPred” (to distinguish the ECPred method from the ECPred dataset, we use “ECPred” to represent the ECPred method, similarly hereinafter), and UDSMProt methods on the DEEPre and ECPred datasets, with, respectively, 3 and 2 levels of prediction tasks. The results indicate that our DAttProt performs generally competitively with the compared methods and significantly outperforms them in the last task level.

Additionally, we make case studies and analyses on the interpretability of our DAttProt method through 351 protein samples and visualize several detailed examples. The case studies prove that the double-scale attention matrix of DAttProt can discover functional sites and regions of the protein sequences indicating their enzymatic functions. All data of this work excluding large model and open-access database files are available at our Github repository.

## 2 Materials and Methods

### 2.1 Datasets and Tasks

All datasets (accessed on 6 January 2021) presented in this study are openly available. Specifically, the datasets and their corresponding tasks applied in this study are illustrated as follows.

The DEEPre database contains 22,166 low-homology enzymes and 22,142 non-enzyme protein sequences that are non-redundant. Enzyme sequences are assigned to 6 main classes and further divided into 58 sub-classes. To avoid data lacking and imbalance problems within all sub-classes, the hierarchical classification is decomposed into 3 independent tasks using a level-to-level strategy. The DEEPre database is available at its website.

The ECPred database consists of 247,527 enzymes and 42,382 non-enzyme (including 55,180 enzymes and 25,333 non-enzymes of UniRef50 Suzek et al. (2015) clusters) protein sequences. The prediction procedure is layered into 5 levels. Levels 0 and 1 are enzyme main classes and non-enzyme prediction. Levels 2, 3, and 4 are detailed sub-class prediction, where enzyme proteins of the UniRef50 clusters are rearranged into training and validation sets for binary classification. Our enzyme class prediction on ECPred covers level 0 (an enzyme and non-enzyme discriminator) and level 1 (6 main-class enzyme selectors). The ECPred database is available at its Github repository.

Both DEEPre and ECPred pick out protein sequences from Swiss-Prot Bairoch and Apweiler (2000), a reviewed protein dataset from the UniProtKB Apweiler et al. (2004) database. The Swiss-Prot database includes 553,941 proteins. Protein samples recorded in Swiss-Prot are non-redundant and manually annotated with high-quality experimental results, computed features, and scientific conclusions. The Swiss-Prot database is available at its website.

In order to enrich the prior proteinic knowledge, our DAttProt model is pre-trained on the full Swiss-Prot protein database. We randomly split raw Swiss-Prot sequences by ratios of 90:5:5 into training, validation, and testing set, referring to UDSMProt Strodthoff et al. (2020). We, respectively, fine-tune our DAttProt model on DEEPre Li et al. (2018) and ECPred Dalkiran et al. (2018) datasets independently on each task level.

We select 351 protein sequences from DEEPre and ECPred datasets for case study. All of these samples are annotated in the Swiss-Prot database and their accession numbers are recorded in the Supplementary Table S2.

### 2.2 DAttProt Method

**FIGURE 1:**
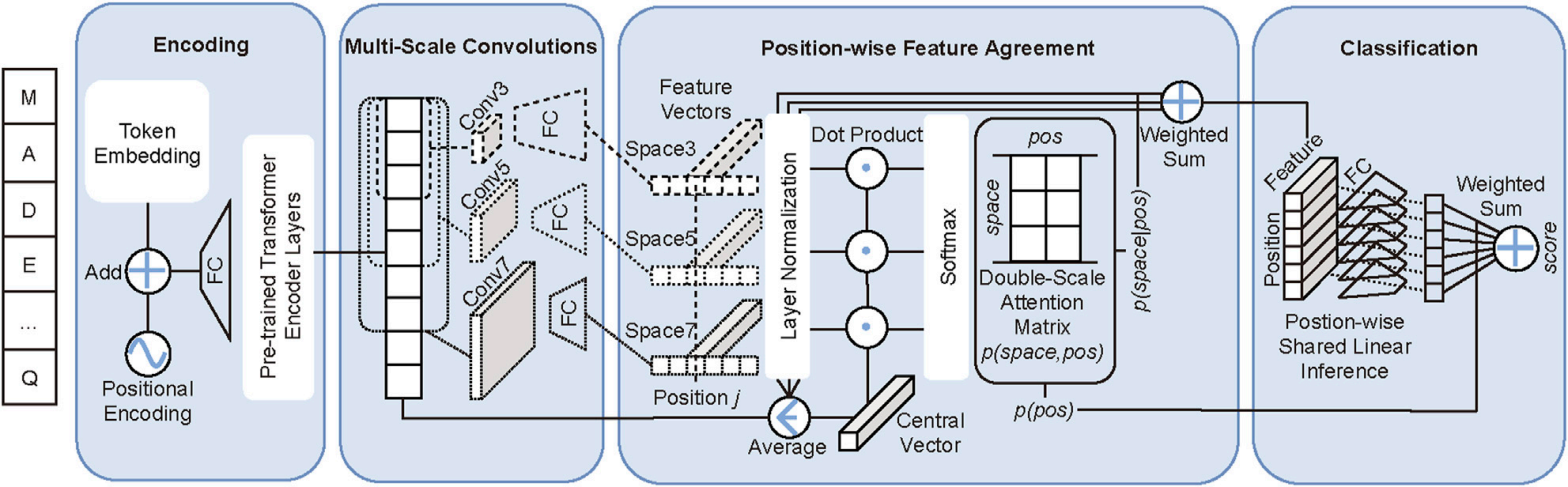
DAttProt model overview. The sequence “MADE … Q” is an example of input protein sequence. FC is the abbreviation of a full connection layer. Conv*k* and Space*k* represent the *k*-kernel–sized convolution and the *k*-sized spatial scale. $p\left(pos\right)$ is the weight vector on the positional scale and $p\left(space,pos\right)$ is the weight matrix on both spatial and positional scales.

As displayed in Figure 1, our double-scale attention model DAttProt is composed of four modules: sequence encoding, multi-scale convolutions, the position-wise feature agreement algorithm, and linear classification.

The core mechanism of our DAttProt method is mixing spatial and positional scale attention. Our DAttProt is based on the thought that a protein sequence classification task allocates primary interest to specialized patterns. These patterns may vary in spatial scale, positional scale, and mixed scales.

To be specific, the multi-scale property of attention-focused patterns lies in the following three aspects:

• Spatial: sub-sequences of various spatial or hierarchical levels contribute diversely to the sequence class predictions. Keywords or core phrases in natural language sentences generally vary in length. In bioinformatic analysis, methods such as *k*-mer search for critical short chains with appointed *k*-scale.
• Positional: positional reliance universally exists in sequences. Empirically, sequential attributes are dominated by critical locations. An essay can be summarized in a few words, and biological molecular functions or structures can be inferred from some specialized regions or sites hiding in the sequence.
• Mixed: positional scales are relative to spatial or hierarchical scales, as crucial patterns for sequence classification may vary in size and locate in scattered sites.

The general motivation of our DAttProt method is to quantify the attention allocated by the classification on both spatial and positional scales. Generally, our DAttProt model processes protein sequential data in three stages: 1) extracting primary global features by constructing Transformer encoder layers transferred from pre-training tasks, 2) double-scale (spatial and positional) relations analysis and spatial scale features merging, and 3) position-wise linear classification inference and prediction summary.

At the second stage above, DAttProt fits a double-scale attention weight matrix of features on both spatial and positional scales. The matrix, in the form of a joint probability distribution, is quantified by our dot-product–based feature agreement algorithm.

Given a sentence, humans tend to roughly group basic units into possible semantic parts according to a prior lexicon and, afterward, search for their logical association and significance Hahn and Keller (2016); Bramall and Higgins (1995). Inspired by these human reading habits, the double-scale attention matrix derives a spatial-scale conditional distribution for feature fusion and a positional-scale margin distribution successively voting for prediction summary.

In Table 1, we list the meanings of important symbols in this study. The detailed realization of the DAttProt model is as follows.

**TABLE 1:** Glossary of important symbols in this article.

Symbol	Meaning	Symbol	Meaning
*x*	Input protein sequence of tokens	*d*_*k*_, *d*_*v*_	Dimensions of key, value features
*l*	Length of input sequence	*F*_*global*_	Output of the Transformer encoders
*S*	Primary sequence embedding of *x*	*S*_*k*_	*k*-th spatial scale or kernel size
*d*	Dimension of an embedding vector	$F_{S_{k}}$	Feature on spatial scale *S*_*k*_
*M*	Number of classes	$F_{S_{k}}^{j}$	$F_{S_{k}}$ ’s feature vector at position *j*
*N*	Number of kernel sizes	$F_{cent}^{j}$	Center vector at position *j*
*h*	Number of heads in Transformer	*A*	Agreement matrix
*pos*	Position	*W*	Double-scale attention matrix
*space*	Spatial scale or kernel size	*F*_*mixed*_	Mixed-scale encoder output
*F*_*in*_	Input of a Transformer encoder	*C*	Positional prediction matrix

#### 2.2.1 Protein Sequence Pre-Processing and Encoding

Generally, amino acids in protein sequence are represented by 22 characters in the alphabet, including 20 standard amino acids and 2 non-standard amino acids. However, there are very few sequences containing ambiguous or unknown amino acids in protein databases JCBN (1984). Token *B* represents Aspartic acid (token *D*) or Asparagine (token *N*) and token *Z* represents Glutamic acid (token *E*) or Glutamine (token *Q*). We replace a token *B* with *D* or *N* randomly with equal probabilities encoding it each time, as well as token *Z*. An unknown amino acid is marked as token *X*, which is treated as equivalent to token *MASK* in our DAttProt due to their generality in context.

Protein data might be variable-length sequences; however, a fixed-length *l* is necessary for mini-batch or full-batch training. We regulate input sequences to *l* length by chopping and padding. To be specific, for data longer than *l*, we cut out *l*-sized sub-sequences randomly. For those shorter, we complement *PAD* tokens at the end, which are encoded as zero vectors without gradients, and their features are also masked to zeros or minuteness constants.

Given an input sequence *x* of length *l*, embedding $S\in R^{l\times f}$ is encoded by the sum of amino acid token embedding (scaled output of a parameterized embedding layer) and cosine positional encoding *PE* Vaswani et al. (2017) as defined by Eq. 1. Settled and regular positional encoding guarantees that well-trained DAttProt is robust for data of variable lengths. *S* is then sent into a pre-trained Transformer encoder block.

$$\begin{array}{cc} PE_{\left(pos,2i\right)} & =\sin\left(pos/10000^{2i/f}\right)\\ PE_{\left(pos,2i+1\right)} & =\cos\left(pos/10000^{2i/f}\right) \end{array}$$ (1)

#### 2.2.2 Transformer Encoders

Each Transformer encoder layer consists of a multi-head self-attention module and a position-wise feed-forward module, both with residual connection and layer normalization. A multi-head attention mechanism is the key component of the Transformer. Given an input sequence coding $F_{in}\in R^{l\times f}$ and *h* groups of project matrices ${\left\{W_{Q}^{i}\in R^{f\times d_{k}},W_{K}^{i}\in R^{f\times d_{k}},W_{V}^{i}\in R^{f\times d_{v}}\right\}}_{i}$ , multi-head attention projects *F*_*in*_ to *h* heads of query, key, and value spaces by Eq. 2. (In our DAttProt, *d*_*k*_ = *d*_*v*_ = *f*/*h*.) Each head proceeds a scaled dot-product attention by Eq. 3 and concatenates *head* ^*i*^ with other heads. Then a matrix *W*_*O*_ projects all the head expressions to the output space.

$$Q^{i},K^{i},V^{i}\leftarrow F_{in}W_{Q}^{i},F_{in}W_{K}^{i},F_{in}W_{V}^{i}$$ (2)

$$head^{i}=\mathrm{softmax}\left(\frac{Q^{i}\cdot {\left(K^{i}\right)}^{T}}{\sqrt{d_{k}}}\right)\cdot V^{i}$$ (3)

Matrix multiplication operations can be effectively deployed in parallel. This is a prominent advantage of the Transformer over RNN-style models, especially in industry.

#### 2.2.3 Multi-Scale Feature Agreement Algorithm

The pre-trained Transformer encoder block extracts global feature $F_{global}\in R^{l\times f}$ from the perspective of the whole sequence. DAttProt employs 1D multi-scale convolutions to capture local reliance and patterns from *F*_*global*_ on *N* assigned spatial scales $\left\{S_{k}\right\}$ . In detail, each convolutional kernel size corresponds to a *S*_*k*_ and step size is uniform 1 to traverse the whole sub-sequential space. Appropriate padding and chopping are operated to hold the output feature-length at *l*. On each spatial scale *S*_*k*_, features are divided by $\sqrt{S_{k}}$ considering their variance expansion after convolutions. Then these local multi-scale features are linearly projected to the same subspace where agreement scores are calculated in the following feature agreement algorithm.

We suppose in a heuristic way that features on different spatial scales but the same positional scale are of great significance only if they are highly identical. For instance, if a feature vector $v_{space=S_{k},pos=j}$ is isolated in a spatial cluster $\left\{v_{pos=j}\right\}$ , it may imply that this *S*_*k*_-sized sub-sequence centering on position *j* is either semantically redundant or incomplete. Inspired by the scaled dot product algorithm of Transformer Vaswani et al. (2017) and the dynamic routing in the capsule networks Sabour et al. (2017), we introduce a feature agreement algorithm as a criterion to quantify each spatial feature’s similarity to the others at its position.

The lengths of feature vectors on the spatial scale are close due to layer normalization Ba et al. (2016) in encoders and scaling after convolutions; thus, their dot products with an average central vector are capable of indicating their similarities (i.e., their agreement levels). Let $F_{S_{k}}\in R^{l\times f}$ be the feature map on the *k*-th spatial scale *S*_*k*_, and $F_{S_{k}}^{j}\in R^{f}$ be $F_{S_{k}}$ ’s feature vector at position *j*. The central vector $F_{cent}^{j}$ and agreement score of $F_{S_{k}}^{j}$ are calculated by Eqs 4, 5.

$$F_{cent}^{j}=\frac{F_{global}^{j}+\overset{N-1}{\underset{k=0}{\sum }}F_{S_{k}}^{j}}{N+1}$$ (4)

$$agree_{S_{k}}^{j}=\frac{F_{S_{k}}^{j}\cdot F_{cent}^{j}}{f}$$ (5)

Specially, we include the original feature map *F*_*global*_ (i.e., the layer-normalized output of Transformer encoders) in the computation of the central vector, where $F_{global}^{j}$ denotes the *j*-th position of *F*_*global*_. With the supervision of *F*_*global*_, the convolutions tend to learn more position-concentrated representations for their receptive fields. All the agreement scores constitute an agreement matrix $A\in R^{N\times l}$ , which is transformed into a double-scale attention matrix $W\in R^{N\times l}$ later. Algorithm 1 describes the feature agreement algorithm process.

Algorithm 1 Feature Agreement Algorithm.

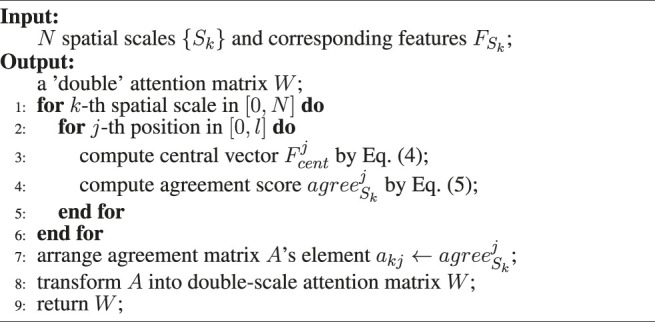

#### 2.2.4 Probabilistic Double-Scale Attention Matrix

To construct an attention matrix *W* in the form of a joint distribution, we flatten the double-scale agreement matrix *A* and reshape it back to size *N* × *l* after Soft-max transformation.

As mentioned above, we interpret *W* as a joint-distribution-formed matrix, whose element *W*_*kj*_ quantifies the significance on the *k*-th spatial and *j*-th positional scale:

$$W_{kj}=p_{att}\left(space=S_{k},pos=j\right).$$ (6)

*W* introduces an aligned attention mechanism on both spatial and positional scales; thus, we name it the double-scale attention matrix. *W* derives the margin-distribution-formed vector *v* by computing row sum. Element *v*_*j*_ of *v* denotes attention score along positional scales:

$$\begin{array}{cc} v_{j}=\overset{N-1}{\underset{k=0}{\sum }}W_{kj} & =\underset{k}{\sum }p_{att}\left(space=S_{k},pos=j\right)\\ & =p_{att}\left(pos=j\right). \end{array}$$ (7)

Then through *W* and *v*, we derive position-wise weights for spatial scale by Eq. 8:

$$\begin{array}{cc} p_{att}\left(space=S_{k}|pos=j\right) & =\frac{W_{kj}}{v_{j}}\\ & =\frac{p_{att}\left(space=S_{k},pos=j\right)}{p_{att}\left(pos=j\right)}. \end{array}$$ (8)

With Eq. 8, we sum up spatial scale features $F_{S_{k}}$ into a mixed feature *F*_*mixed*_ by Eq. 9, in which $F_{S_{k}}^{j}\in R^{f}$ represents the *j*-th location of spatial feature $F_{S_{k}}$ .

$$F_{mixed}=\overset{N-1}{\underset{k=0}{\sum }}\overset{l-1}{\underset{j=0}{\sum }}\frac{W_{kj}}{v_{j}}\cdot F_{S_{k}}^{j}$$ (9)

As discussed in 2.2.3, the isolated spatial feature (i.e., a feature vector with a large angle from the central vector in space) is not significant at its position and obtains low agreement weight. Therefore, through Eq. 9, *F*_*mixed*_ pays more attention to informative features on each positional scale. Even if an isolated vector contains part of a meaningful pattern, convolutions with 1 step size will recapture a pattern of greater integrity at another position.

#### 2.2.5 Linear Classifier

DAttProt makes inferences from position-wise to global-wise. We map the mixed-scale encoder output features $F_{mixed}\in R^{l\times f}$ to a probabilistic space of *M* classes through linear (one-layer full connection) and Soft-max transformations, yielding a positional prediction matrix $C\in R^{l\times M}$ . On the *j*-th positional scale, the element of *C* is a prediction score by Eq. 10:

$$C_{dj}=p\left(x\in \text{class }d|pos=j\right),$$ (10)

where *d* = 0, 1, … , *M* − 1.

Finally, we conclude the positional prediction from *C*, working out the probability that input sequence *x* belongs to class *d* by Eq. 11.

$$\begin{array}{cc} p\left(x\in \text{class }d\right) & =\overset{l-1}{\underset{j=0}{\sum }}C_{dj}\cdot v_{j}\\ & =\underset{j}{\sum }p\left(x\in \text{class }d|pos=j\right)\cdot p_{att}\left(pos=j\right) \end{array}$$ (11)

### 2.3 Pre-Training and Fine-Tuning

Self-supervised pre-training boots up DAttProt’s encoder module as a language model. The pre-training task of DAttProt is similar to the Masked Language Model (Masked LM) task in BERT (Devlin et al., 2018). DAttProt learns to predict 15% tokens of input sequence at random positions (excluding unknown tokens *X*), where each input token is randomly replaced by *MASK* token by 80% or another token in the amino acid vocabulary by 10%. The language model additionally trains a simple position-wise linear classifier to complete token predictions. Similar to the original Transformer, the warm-up strategy and learning rate decay are applied in pre-training.

We fine-tune the encoders, multi-scale convolutions, and linear classifier of DAttProt for the downstream tasks on the DEEPre and ECPred datasets. For each task level on each dataset, an individual DAttProt model is fine-tuned. We minimize the cross-entropy loss using an AdamW optimizer (Loshchilov and Hutter, 2018) both in pre-training and fine-tuning.

### 2.4 Hyper-Parameter Settings

All hyper-parameters of 6-layer DAttProt can be looked up at Github.

The pre-training model unifies the input sequences to 512 tokens long. The batch size is 32 due to the GPU memory restriction (occupying about 14G for 6 layers and 6G for 3 layers), and the max pre-training epoch is 300. The dimensions of initial embedding features and hidden states in 8-headed Transformer encoders are 16 and 512. Dropout probabilities are globally set to be 0.1. We mask the *PAD* tokens to avoid their participation in the multi-head self-attention.

As described in section 2.3, we pre-train 6-layer Transformer encoders with a schedule of the learning rate. We set the warm-up iteration to be 50,000 where the learning rate reaches the maximum of 0.00005 (0.0001 for the 3-layer model).

The fine-tuned model applies convolutions of 5, 10, and 20 kernel sizes according to the common scales of annotated motif features (the lengths of most motifs recorded in the Swiss-Prot database are less than or equal to 20). For each scale of the convolution, the number of output channels is 16 times the kernel size, which is reduced to 16 after activation and a linear transformation. The batch size is set to 128, and the max fine-tuning epoch is 100 (50 for level 2).

The learning rate is set to be 0.00001 at the beginning of fine-tuning. Then, it is multiplied by 0.95 after every hundred iterations. We add a regularization with 0.0001 weight decay to avoid over-fitting.

## 3 Results

### 3.1 Compared Methods

In this study, we compare our DAttProt with three methods on two datasets described in section 2.1. The first compared method is “DEEPre” Li et al. (2018) proposed by Li et al., which encodes protein sequences and features of the PSSM matrix in both sequence length–dependent and –independent ways. The second method is “ECPred” Dalkiran et al. (2018) proposed by Dalkiran et al., which refers to ensemble learning and trains three types of predictors for each EC class. “DEEPre” and “ECPred” methods are only deployed in their corresponding datasets, set as a baseline for each dataset. Third, we compare with UDSMProt Strodthoff et al. (2020) proposed by Strodthoff et al. on both datasets. UDSMProt separately pre-trains one forward, one backward, and one bi-direction AWD-LSTM, applying self-supervised learning. UDSMProt additionally trains a CNN baseline model, taking sequences and PSSM features as inputs. However, we ignore this baseline model due to possible data leakage problems related to PSSM features.

We pre-train two DAttProt encoder modules that are different in the amount of Transformer encoder layers, one with 3 layers and another with 6. For the DEEPre dataset, we use 5-fold cross-validation to measure the accuracy according to the baseline method “DEEPre” and the compared method UDSMProt. We separately train predictors for levels 0, 1, and 2 and calculate the mean accuracy of data branches in each level. For the ECPred dataset, samples are divided into training and test sets referring to the “ECPred” baseline method. We train predictors for levels 0 and 1 and take the average macro-*F*_1_ score as the performance measure.

### 3.2 Analysis for Comparison Results

For DEEPre and ECPred datasets, the level-wise prediction performance results of our DAttProt and the compared methods mentioned above are respectively listed in Table 2, 3.

**TABLE 2:** Performance results on DEEPre dataset.

Method		Task level/acc. (var. × 10^3^)		
		0	1	2
“DEEPre”		**0.883 (0.012)**	0.826 (0.017)	0.436 (0.135)
UDSMProt	Forward	0.867 (0.015)	0.816 (0.020)	0.753 (0.075)
	Backward	0.861 (0.017)	0.834 (0.022)	0.739 (0.083)
	Bi-direction	0.871 (0.010)	0.845 (0.020)	0.781 (0.066)
DAttProt	3-layer	0.858 (0.016)	0.821 (0.019)	0.736 (0.080)
	6-layer	0.877 (0.018)	**0.859 (0.020)**	**0.788 (0.071)**

The results of level 2 are first calculated by the average of 6 sub-class classification results. All the results are average values calculated by 5 times of experiments on 5 folds and bold values are the best results of each task level (similarly hereinafter). acc: accuracy var: variance.

**TABLE 3:** Performance results on ECPred dataset.

Method		Task level					
		0			1		
		*P*	*R*	*F*_1_ (var. × 10^3^)	mac.-*P*	mac.-*R*	mac.-*F*_1_ (var. × 10^3^)
“ECPred”		0.972	0.965	0.964 (0.008)	0.970	0.948	0.963 (0.010)
UDSMProt	Forward	0.967	0.958	0.955 (0.013)	0.958	0.926	0.933 (0.026)
	Backward	0.969	0.962	0.967 (0.015)	0.957	0.933	0.935 (0.022)
	Bi-direction	0.979	**0.966**	**0.968 (0.011)**	0.963	0.940	0.944 (0.025)
DAttProt	3-layer	0.962	0.934	0.944 (0.016)	0.953	0.904	0.925 (0.023)
	6-layer	**0.983**	0.960	0.965 (0.012)	**0.977**	**0.951**	**0.967 (0.016)**

All the results are average values calculated by 5 times of experiments and bold values are the best results of each task level (similarly hereinafter). P: Precision R: Recall var: variance mac-: macro-

As shown in Table 2, 3, the 6-layer DAttProt is competitive with the baseline and the AWD-LSTM–based UDSMProt predictors. The baseline methods, “DEEPre” and “ECPred”, deploy the PSSM matrix and count the distribution of position-wise amino acids from the Swiss-Prot database. Our DAttProt model accepts only raw sequences and predicts as precisely as the baseline methods. This result indicates that the Transformer encoder module can extract implicit distribution information of the sequences. Especially, DAttProt and UDSMProt outclass the “DEEPre” method on level 2 of the DEEPre task, which reflects the reliability of pre-trained methods on complex tasks.

However, only average accuracy and macro-*F*_1_ are compared in comparison experiments. We record more results to measure the performance of our DAttProt method. As shown in Table 5, we list the average accuracy (acc.), Precision (P), Recall (R), and *F*_1_ (macro-*F*_1_ for levels 1 and 2) of DAttProt on both DEEPre and ECPred tasks.

On the last task level of each dataset, we conduct unpaired and one-tailed heteroscedastic Student’s t-test Walpole (2006) on the performance of 6-layer DAttProt over compared methods. On level 2 of the DEEPre dataset, our 6-layer DAttProt gets 0.0014 p-value over bi-direction UDSMProt and p-values less than 0.0001 over other compared methods. On level 1 of the ECPred dataset, 6-layer DAttProt gets 0.0445 p-value over the ‘ECPred’ method and p-values less than 0.0001 over other compared methods. All the p-values are less than 0.05, showing that our 6-layer DAttProt method performs significantly better than the compared methods on the task levels above with significance level over 95%. In Table 6, we list the p-values between 6-layer DAttProt and each compared method on the last task level of each dataset.

Vaswani et al. (2017) proved that the deeper the Transformer encoders, the more global relations the output features include. Compared with the 6-layer DAttProt, the 3-layer DAttProt performs globally weaker, especially on ECPred tasks. This result emphasizes the contribution of global sequential reliance to the predictions.

### 3.3 Case Study and Interpretability Analysis

According to section 2.2.4, DAttProt computes an intermediate matrix called the double-scale attention matrix. We assume that this matrix learns to give high attention scores to motif sites of protein sequences (on the positional scale) and the kernel size closest to the motif length (on the spatial scale). To practically verify our hypothesis, we select protein samples from DEEPre and ECPred datasets whose motif features are annotated in the Swiss-Prot database and input them into the fine-tuned DAttProt model. We fetch out the positional and spatial scale weights of the double-scale attention matrix to study its interpretability.

**FIGURE 2:**
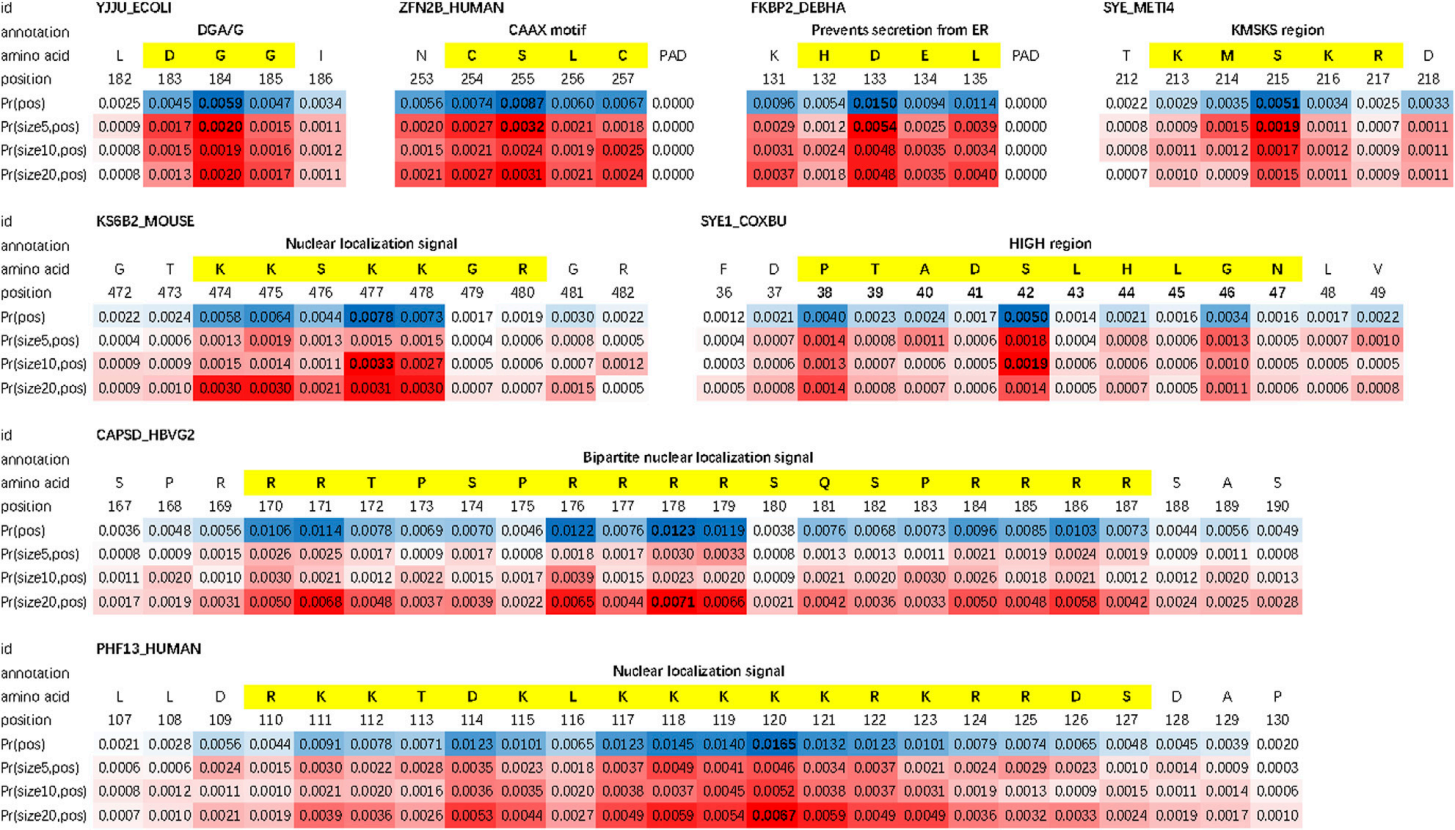
: Examples of matched motifs in different sizes. Their spatial sizes vary from 3 to over 15. $\mathrm{Pr}\left(pos\right)$ is the marginal weight vector on the positional scale and $\mathrm{Pr}\left(sizeK,pos\right)$ is the weight vector of *K*-sized sub-sequences on the positional scale (similarly hereinafter).

We evaluate the double-scale attention matrix of our DAttProt with over 300 annotated protein samples. For one annotation of a functional site or region, if its length is closest to the kernel size with the highest attention and its middle position is given a weight more than 1.5 times that of the average weight, we consider it as a match by the matrix. For each sequence, we sort the matched areas by their double-scale attention scores in descending order and select the first 16 (if any) of them. After matching over 100 motifs in these enzymes, we conclude that our model is able to locate the positions and sizes of functional protein motifs. As shown in Figure 2, the double-scale attention matrix matches some high-probability regions with variable-sized annotated motifs that are closely related to their enzyme functions or analysis. For example, the CAAX motif found at the end of protein ZFN2B_HUMAN (accession number Q8WV99) can be applied to molecular dynamics simulations Sousa et al. (2013). The slightly longer motif, nuclear localization signals (NLSs), is involved with protein functions in the cell nucleus Schwab (2009).

**FIGURE 3:**
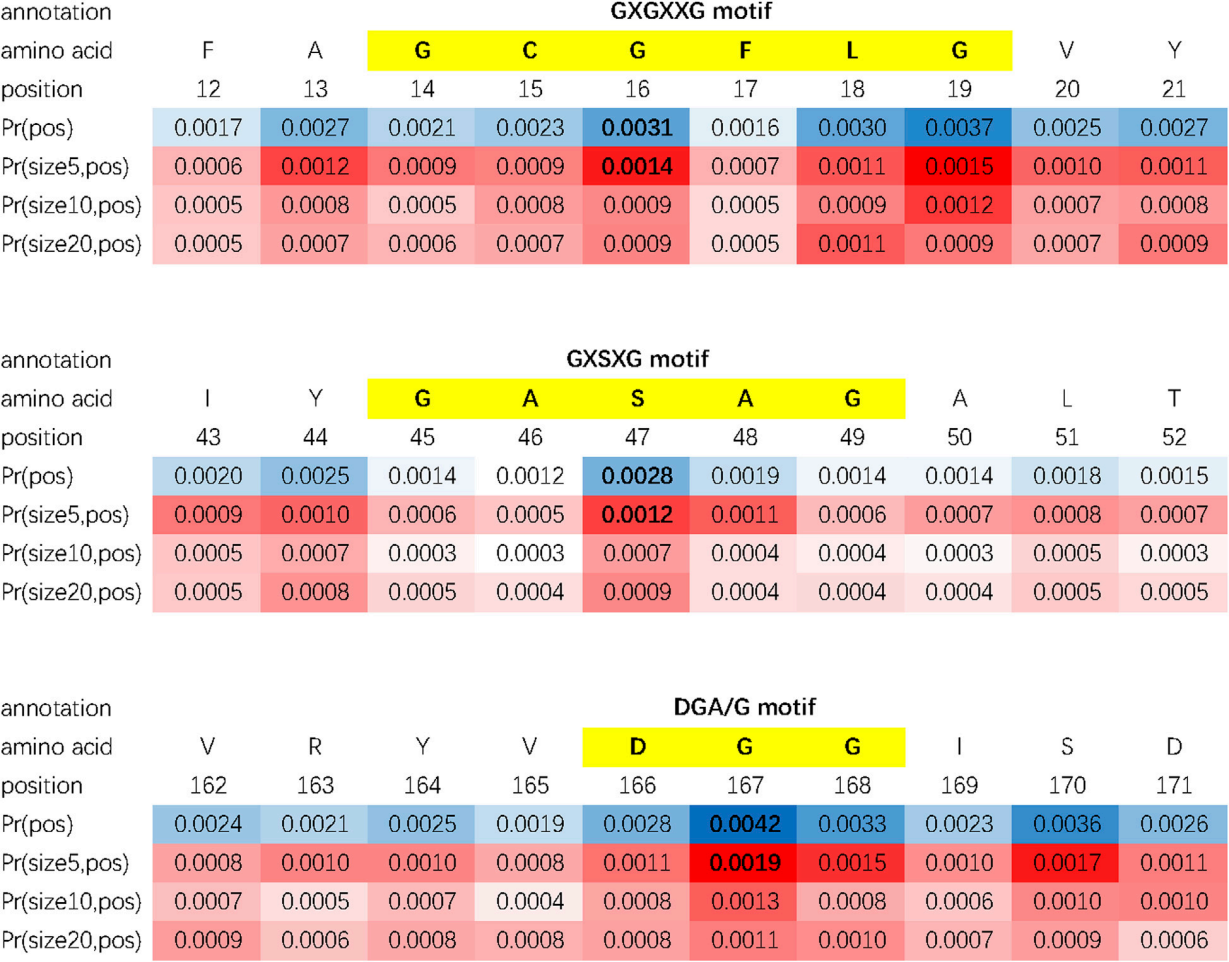
Three matched motifs belonging to the PNPLA domain.

Moreover, we find that for each protein sequence, the double-scale attention matrix does not concentrate all the weights on one region but tries to discover as many sub-sequences as possible. As an example, three motifs (shown in Figure 3) are matched in protein FKBP2_DEBHA (accession number Q6BP84). These three motifs are all features of the patatin-like phospholipase (PNPLA) domain, which implies lipase and transacylase properties of the containing proteins Sigrist et al. (2002). We list all matched protein motifs and the corresponding sequence ids in Supplementary Table S2.

**FIGURE 4:**
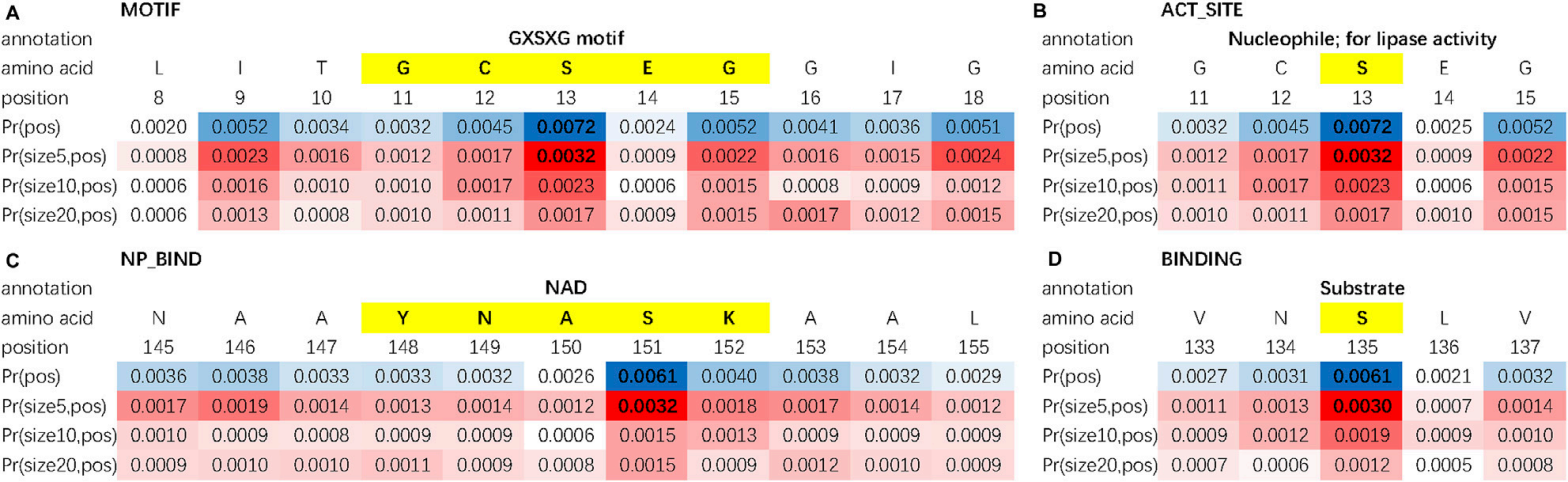
Matched sites and regions of AYR1_SCHPO. **(A)** Motif. **(B)** Amino acid involved in the enzymatic activity. **(C)** Binding site. **(D)** Extent of a nucleotide phosphate-binding region.

Besides protein motifs, some other key sites or regions can be found by using the double-scale attention matrix. In Figure 4, we investigate the double-scale attention matrix of the protein AYR1_SCHPO (accession number Q09851) and discover that its enzymatic activity site of lipase, substrate binding site, and nucleotide phosphate-binding region [referring to the Swiss-Prot database Bairoch and Apweiler (2000)] are also given high attention scores by DAttProt.

In conclusion, our DAttProt observes variable-sized protein functional sites or regions and allocates high attention weights to the central position and the scale closest to the motif size. The statistical data of matched motifs in case study are listed in Table 4.

**TABLE 4:** Statistical data of matched motifs in case study.

Field	Count
Protein samples	351
Total motif matches	483
Matched motif types	113
Motif size in range $\left[1,8\right)$	370
Motif size in range $\left[8,15\right)$	57
Motif size in range $\left[15,25\right)$	50

For full data please refer to the Supplementary Table S2

**TABLE 5:** Detailed performance indexes of DAttProt.

Task	Level	Branch	3-layer				6-layer			
			acc	P	R	*F*_1_	acc	P	R	*F*_1_
DEEPre	0	Proteins	0.85	0.85	0.79	0.80	0.87	0.86	0.80	0.81
	1	Enzymes	0.82	0.70	0.64	0.59	0.85	0.84	0.80	0.80
	2	Oxidoreductases	0.61	0.56	0.46	0.48	0.72	0.70	0.62	0.63
		Transferases	0.70	0.56	0.51	0.53	0.76	0.74	0.71	0.72
		Hydrolases	0.72	0.42	0.40	0.39	0.80	0.59	0.54	0.55
		Lyases	0.78	0.61	0.50	0.53	0.81	0.76	0.73	0.74
		Isomerases	0.81	0.86	0.77	0.79	0.83	0.88	0.78	0.80
		Ligases	0.80	0.62	0.56	0.58	0.81	0.70	0.64	0.65
ECPred	0	Proteins	0.91	0.96	0.93	0.94	0.94	0.98	0.96	0.97
	1	Enzymes	0.92	0.95	0.90	0.93	0.96	0.98	0.95	0.97

**TABLE 6:** p-values of the unpaired and one-tailed heteroscedastic Student’s t-test on 6-layer DAttProt over compared methods on the last task level of each dataset.

Dataset	Compared methods					
	“DEEPre”	“ECPred”	UDSMProt			3-layer DAttProt
			Forward	Backward	Bi-direction	
DEEPre Lv.2	3.955e-57	-	6.114e-19	2.052e-24	1.387e-3	5.360e-26
ECPred Lv.1	-	4.446e-2	3.710e-6	3.027e-6	4.296e-5	5.890e-7

For each method, the calculated *p*-values are based on 25 results of DEEPre and 5 results of ECPred. We use *MeN* to represent *M* × 10^*N*^ in the form of scientific notation

## 4 Conclusion and Discussion

Enzyme class prediction is an important task for enzyme protein classification and annotation. The task predicts the EC number of a protein sequence which identifies the enzyme function in biological metabolism. Previous computational methods of enzyme class prediction rely on either prior distribution information or deep learning architectures that ignore the significance of biological interpretability.

In this study, we propose an effective and interpretable double-scale attention method, DAttProt, for enzyme class prediction tasks. The performance and interpretability of DAttProt have been proved in our experiments. The pre-trained Transformer encoders adequately extract the global amino acid relations from raw protein sequences, offering an alternative to the traditional assistant features such as the PSSM matrix. Case study illustrates that the double-scale attention matrix calculated during fine-tuning discovers biologically meaningful sub-sequences such as functional motifs of enzymes by allocating attention weights.

Previous deep learning methods for predicting enzyme classes extract high-level protein features and make classification inference in a black box. The techniques and architectures they apply are proven in practice and offer remarkable results such as high accuracy. However, they cannot produce a convincing theory of prediction or the underlying basis with biological and statistical knowledge. Compared with these methods, our DAttProt method goes one more step for biological interpretability. Two points considered in our model design are the relationships between pairs of amino acids and possible contributions of functional sites and regions in the protein sequence.

In specific, our DAttProt method applies a self-attention mechanism to quantify position-wise relations between each pair of amino acids in parallel for sequence embedding. Then multi-scale convolutions extract local features which are the basic representations of sub-sequences in the protein. Our core idea lies in the design of the feature agreement algorithm and its production—the double-scale attention matrix. As a result, DAttProt not only makes accurate classification results but also offers straightforward evidence supporting the prediction. Furthermore, DAttProt may provide some new discovery about functional sites and regions in enzyme proteins.

Although DAttProt possesses the above advantages over previous methods in enzyme class prediction tasks, it has a few limitations. The model randomly chops the longer protein sequences to a fixed length and might drop some information of these samples. The amount and sizes of multi-scale convolution kernels need to be manually assigned according to common motif lengths.

For further research, pre-processing and dimension reduction of long sequence inputs will be introduced for better model performance. To better processing and encoding long sequences, some advanced methods Chen et al. (2018); Child et al. (2019); Goyal et al. (2020); Dai et al. (2020); Roy et al. (2021) might be applied to improve or replace Transformer encoders. To further reduce manual intervention during fine-tuning, we consider replacing multi-scale convolutions with a self-adapting convolution module Su et al. (2019); Chen et al. (2020); Lioutas and Guo (2020) that can flexibly adjust kernel size for each input sequence or amino acid by inference. Furthermore, the heuristic feature agreement algorithm described in section 2.2.3 can be improved in theory to be stricter and capable of recognizing multi-functional sub-sequences.

## Data Availability

The original contributions presented in the study are included in the article/Supplementary Material, further inquiries can be directed to the corresponding author. Source codes of this work are available at https://github.com/zhanglabNKU/DAttProt
